# Characterization of Lithuanian Tomato Varieties and Hybrids Using Phenotypic Traits and Molecular Markers

**DOI:** 10.3390/plants13152143

**Published:** 2024-08-02

**Authors:** Audrius Radzevičius, Jūratė Bronė Šikšnianienė, Rasa Karklelienė, Danguolė Juškevičienė, Raminta Antanynienė, Edvinas Misiukevičius, Aurelijus Starkus, Vidmantas Stanys, Birutė Frercks

**Affiliations:** Lithuanian Research Centre for Agriculture and Forestry, Institute of Horticulture, LT-54333 Babtai, Kaunas District, Lithuaniaedvinas.misiukevicius@lammc.lt (E.M.);

**Keywords:** genetic resources, microsatellite, phenotypic traits, *Solanum lycopersicum*

## Abstract

The aim of this study was to evaluate phenotypic traits and genetic diversity of the 13 tomato (*Solanum lycopersicum* L.) varieties and 6 hybrids developed at the Institute of Horticulture Lithuanian Research Centre for Agriculture and Forestry (LRCAF IH). For the molecular characterisation, seven previously published microsatellite markers (SSR) were used. A24 and 26 alleles were detected in tomato varieties and hybrids, respectively. Based on the polymorphism information content (*PIC*) value, the most informative SSR primers for varieties were TMS52, TGS0007, LEMDDNa and Tom236-237, and the most informative SSR primers for hybrids were SSR248 and TMS52. In UPGMA cluster analysis, tomato varieties are grouped in some cases due to genetic relationships, as the same cluster cultivars ‘Viltis’ (the parent of cv. ‘Laukiai’) and ‘Aušriai’ (the progeny of cv. ‘Jurgiai’) are present. The grouping of all hybrids in the dendrogram is related to the parental forms, and it shows the usefulness of molecular markers for tomato breeding, as they can be used to trace the origin of hybrids and, eventually, varieties accurately. The knowledge about the genetic background of Lithuanian tomato cultivars will help plan targeted crosses in tomato breeding programs.

## 1. Introduction

The cultivated tomato (*Solanum lycopersicum* L.) is the second most consumed vegetable worldwide and a major source of phytonutrients to human health. Huge variations can be found in the cultivated germplasm in terms of fruit colour (white, yellow, orange, pink, red, brown, and green), fruit shape (flattened, oblong, circular, cylindric, elliptic, cordate, ovate, pyriform, and obcordate), and fruit size (very little, small, medium, large, and very large) [1]. Owing to their economic significance, breeders have created many new tomato varieties each year, with the hybrid tomato being one of the most popular. The F1 hybrid cultivars are known for their high yields and occasionally blend the quirky qualities of heirloom tomatoes with the hardiness of regular commercial tomatoes [2]. The associated health impacts of tomatoes on cancer, neurodegenerative and cardioprotective effects, diabetes, and the immune system were recently reviewed [3].

Genetically, genomically, and breeding-wise, tomatoes are a highly studied agricultural crop. The tomato is one of the first crop plants for which a genetic linkage map was created. Currently, there are several molecular maps based on crosses between the cultivated and various wild species of tomato [4]. Based on an *L. esculentum* × *L. pennellii* hybrid, the high-density molecular map was created, containing over 2200 markers with an average distance between markers of less than 1 cM and an average of 750 kbp per cM [4,5].

Variety selections under similar growing conditions can achieve up to two times higher yields from the same plot area and significantly increase the economic efficiency of tomato production. A thorough understanding of the phylogenetic relationship of tomato varieties and the parentage of tomato hybrids is essential to identifying and selecting tomato lines for further variety breeding [6,7,8].

Breeders could greatly benefit from using molecular tools like molecular markers to help them create new elite cultivars. Molecular markers like simple sequence repeats (SSRs) are frequently used for cultivar fingerprinting and genetic diversity studies of tomatoes [9,10,11], and some of them were found to be linked to disease resistance [12,13] or salt tolerance [14,15]. Because SSR markers are highly repeatable, co-dominant (allowing for the identification of both parental alleles), and have broad coverage across the tomato genome, they help identify tomato hybrids. This makes them an effective tool for choosing and preserving hybrid lines with desired features in tomato breeding projects and to avoid misnaming varieties [16]. The new SSR markers are constantly being developed to link them to abiotic or biotic stress [17,18].

In the Lithuanian Research Centre for Agriculture and Forestry Institute of Horticulture (LRCAF IH), a large collection of over 500 different tomato genotypes was created, which could be used to develop new tomato varieties and hybrids. Before selecting genotypes for further breeding, evaluating the entire tomato collection by quantitative and qualitative attributes is important. Hence, the objective of this study was to evaluate 28 tomato genotypes developed in Lithuania using phenotypic traits and molecular markers. This is important in tomato breeding and selecting parental forms, which stand out in their valuable biological properties and transfer them to the new hybrids.

## 2. Materials and Methods

### 2.1. Plant Material

**Figure 1:**
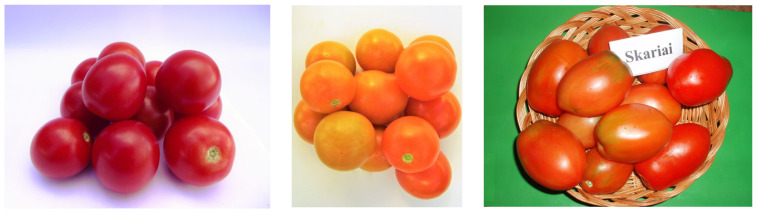
Lithuanian tomatoes. From the left: ‘Adas’ hybrid, ‘Auksiai’ hybrid and ‘Skariai’ variety.

In total, 28 genotypes of tomato were studied; 13 tomato varieties and 6 hybrids were developed at LRCAF IH, and the 9 parental forms of hybrids were investigated (Table 1; Figure 1). Tomato seeds were sown in wooden boxes, and later sprouts with two real leaves were planted into polypropylene pots (pot volume 1.13 L, height 13.9 cm and diameter 11.6 cm). In total, 336 seedlings were grown (12 seedlings per one genotype). Grown seedlings were transplanted to a permanent place of growth in the natural soil in the unheated greenhouse and grown according to tomato-growing technology adopted by the Institute of Horticulture [19,20].

### 2.2. Phenotypic Traits Evaluation

Morphological tomato features of different varieties were recorded using The International Union for the Protection of New Varieties of Plants (UPOV) guidelines [21]. The average fruit mass was obtained by weighing all tomato fruits from four plants in three replications (twelve plants) and dividing it by the number of weighted fruits. The average fruit number was counted for four plants in three replications (twelve plants). Plant growth habit, leaf division of blade, pedicel abscission layer, fruit shape, time of tomato maturity, fruit green shoulder, colour at maturity and amount of locules were observed according to “UPOV” descriptions. Four plants in three replications (twelve plants) were selected for observation. Tomato fruit firmness was measured using the texture analyser “TA.XTPlus” (Stable Micro Systems, Godalming, UK). For each test, every tomato was punctured in three specific positions around the equatorial area, approximately 120° between them and perpendicular to the stem-bottom axis. The obtained data were processed by “Texture Exponent 32” software. The firmness was expressed as the peak force and recorded in Newtons (*N*). 

### 2.3. DNA Extraction

Young leaves from 10 seedlings of each genotype studied (280 samples in total) were collected, flash-frozen with nitrogen and kept at −70 °C until further analysis. DNA was extracted using a modified [22] Cetyltrimethyl ammonium bromide (CTAB) method [23]. DNA was dissolved in 100 μL of TE buffer. The extracted DNA was quantified using a NanoDrop spectrophotometer (Implen, München, Germany) and normalised to a concentration of 300 ng/μL. 

### 2.4. SSR Analysis

Polymerase chain reaction (PCR) was performed using 14 previously published SSR primers with Eppendorf Mastercycler x50a (Eppendorf, Hamburg, Germany). PCR amplifications were performed with 10 μL total volume of the reaction mixture, consisting of (300 ng/μL) DNA, 0.2 mM of each primer, 25 mM of MgCl, 2 mM of dNTP, 10 × buffer, 10 mM of DTT, 1% PVP, and 500 U Taq DNA polymerase (Thermo Scientific, Waltham, MA, USA). The PCR was performed with the conditions as follows: Initial denaturation at 94 °C for 10 min followed by five cycles at 94 °C for 30 s, X °C for 45 s and 72° for 1 min with touchdown procedure at primer annealing step (−1 °C in each cycle). Then, 30 cycles at 94° for 45 s, Y °C for 45 s and 72° for 1 min, with a final extension step of 10 min at 72°, where Y is the appropriate annealing step of primer and X = Y + 7 is the initial temperature for each primer by the touchdown procedure. The agarose gel was used to pre-screen the SSR primers. Eight out of ten used SSR primer pairs showed amplicons, and the forward primer was labelled with a fluorescence dye FAM, NED or VIC (Table 2). Capillary electrophoresis was performed with ABI 3130 Genetic Analyzer (Applied Biosystems, Foster City, CA, USA) using a standard (GeneScan 500LIZ).

### 2.5. Genetical Data Analysis

Primary data were analysed using the software GeneMapper v.4.0 (Thermo Fisher Scientific Inc., USA). The frequency of alleles, expected (*H_e_*) and observed heterozygosity (*H_o_*), and the polymorphism information content (*PIC*) were calculated for each SSR primer pair using PowerMarker software v.3.25 [24]. The informativeness of microsatellite loci was established following [25], where *PIC* > 0.5 was considered highly informative, 0.5 > *PIC* > 0.25 was reasonably informative, and *PIC* < 0.25 was slightly informative. The phylogenetic tree was constructed using the “UPGMA” tree method within DARwin Software 6.0.0.021 [26]. To test the reliability of the dendrograms, a bootstrap analysis with 10,000 replications was performed within software DARwin.

**Table 2:** Tomato SSR primer pairs.

No.	Name of Primer	Reference	Annealing Temp. °C	DNA Sequence
1.	SSR-47	[26]	56	NED-TCCTCAAGAAATGAAGCT CTG A
				CCTTGGAGATAACAACCACAA
2.	Tom236-237	[26]	56	NED-GTTTTTTCAACATCAAAGAGCT
				GGATAGGTTTCGTTAGTGAACT
3.	LEat014	[26]	n.a.	VIC-TGTGTTGCGTCATTACCACTAAAC
				CCCAACCACCAATACTTTCC
4.	Tom-59-60	[27]	48	VIC-CACGTAAAATAAAGAAGGAAT
				TAACACATGAACATTAGTTTGA
5.	TMS52	[28]	55	FAM-TTCTATCTCATTTGGCTTCTT C
				TTACCTTGAGAATGGCCTTG
6.	SSR248	[11]	57	NED-GCATTCGCTGTAGCTCGTTT
				GGGAGCTTCATCATAGTAACG
7.	LEMDDNa	[29]	53.5	VIC-ATTCAAGGAACTTTTAGCTCC
				TGCATTAAGGTTCATAAATGA
8.	TGS0007	[30]	51.5	FAM-GTGGATTCACTTACCGTTACAAGTT
			52.4	CATTCGTGGCATGAGATCAA

## 3. Results

### 3.1. Phenotypic Trait Evaluation

According to the phenological data obtained in this research, it was established that the average fruit mass had varied from 60 g (var. ‘Balčiai’, ‘Svara’, ‘Laukiai’) up to 160 g (var. ‘Milžinai’) in all investigated varieties and from 38 g (‘Adas’) up to 78 g (‘Sveikutis’) in hybrids (Table 3). It was observed that varieties with small fruits had produced more fruits on one plant with 2–4 fruit locules, and tomato varieties and hybrids with bigger fruits had a smaller number of tomato fruits on the one plant, and their fruits had more locules (from 4 up to 6 and more). Also, it was established that the vining of four tomato varieties (‘Skariai’, ‘Milžinai’, ‘Dručiai’, ‘Rutuliai’) and four hybrids (‘Sveikutis’, ‘Adas’, ‘Auksiai’, ‘Arvaisa’) was indeterminate, and other bushes of the remaining nine varieties were determinate. Three out of all investigated varieties and hybrids (‘Balčiai’, ‘Jurgiai’, ‘Milžinai’) had bipinnate leaf division of blade and only one variety ‘Neris’ had no peduncle abscission layer.

Most of the investigated tomatoes had slightly flattened fruits. Only varieties ‘Skariai’ and hybrid ‘Ainiai’ fruits were obovoid. Fruits of varieties ‘Svara’, and hybrids ‘Adas’ and ‘Auksiai’, were round, and varieties ‘Jurgiai’ were circular. 

The majority of the investigated Lithuanian varieties were average early maturing. Only varieties ‘Viltis’, ‘Laukiai’ and ‘Aušriai’ were intended for early harvest, and one variety ‘Svara’ was intended for late harvest. Meanwhile, Lithuanian tomato hybrids were only average early (‘Sveikutis’, ‘Ainiai’, ‘Arvaisa’) or early (‘Pirmutis’, ‘Adas’, ‘Auksiai’). 

One tomato hybrid, ‘Auksiai’, had orange-coloured fruits, and all the remaining tomato fruits of investigated varieties and hybrids were red-coloured. Still, some of them (‘Skariai’, ‘Milžinai’, ‘Viltis’, ‘Laukiai’, ‘Vytėnų didieji’, ‘Slapukai’) had green shoulders before maturity.

The fruits of tomato varieties ‘Laukiai’, ‘Jurgiai’, and hybrid ‘Auksiai’ were very soft and had weak fruit firmness. Meanwhile, the fruits of varieties ‘Balčiai’, ‘Vytėnų didieji’, ‘Dručiai’, and hybrid ‘Ainiai’ had strong fruit surface textures (Table 3).

### 3.2. Genetic Analysis

#### 3.2.1. Molecular Fingerprints of Tomato Genotypes

DNA fragments were generated with all eight SSR primer pairs used in this study. Only one monomorphic fragment was generated with primer Tom 59-60; therefore, it was not used for further analysis. All the tomato varieties were homozygous within the seven remaining SSR primer loci (Table 4). 

However, two heterozygotic fragments were generated with some SSR primer pairs (Table 5) in the investigated tomato hybrids. In some cases, hybrids of the same parental forms were found to have three different loci variants, either heterozygous or homozygous from one or the other parental form. The hybrid ‘Arvaisa’ had the largest number of such loci (five loci). By excluding these multiple loci, the number of homozygous loci in hybrids varied from two (‘Arvaisa ‘) to five (‘Auksiai’), and the highest heterozygotic number was observed in hybrid ‘Pirmutis’. All parental forms of the hybrids were monomorphic in all SSR loci. No SSR markers were found to be linked to any phenotypic trait either for varieties or hybrids.

#### 3.2.2. SSR Primer Informativeness

In total, 25 polymorphic alleles were identified in tomato varieties and 26 in tomato hybrids, respectively (Table 6). The number of alleles with each primer pair ranged from 2 to 5 in all investigated varieties and hybrids, but the average value for hybrids was slightly higher (3.71) compared with varieties (3). The allelic range in all SSR loci was higher for hybrids, except loci TMS52, where the range was more comprehensive in varieties than in hybrids. So, all varieties were homozygous; therefore, the observed heterozygosity value (*H_o_*) in all loci was 0. The expected heterozygosity (*H_e_*) values for varieties ranged from 0.14 to 0.72, with an average of 0.51. *H_e_* ranged from 0.33 to 0.66 for tomato hybrids, with an average value of 0.51. Despite the average values being equal in four loci, Tom236-237, LEMDDNa, TMS52 and TGS0007, the *H_e_* values were higher for varieties. Polymorphism information content (*PIC*) for varieties ranged from 0.13 to 0.68, with an average of 0.47 (Table 6). The primer pairs TMS52, TGS0007, LEMDDNa and Tom236-237 were highly informative (*PIC* > 0.5) according to the informativeness of Botstein [25]. The *PIC* value for tomato hybrids ranged from 0.31 to 0.61, with an average of 0.45, and there were only two highly informative SSR primers, SSR248 and TMS52, for hybrids. 

#### 3.2.3. Genetic Diversity and Relationships of Tomato Varieties and Hybrids

**Figure 2:**
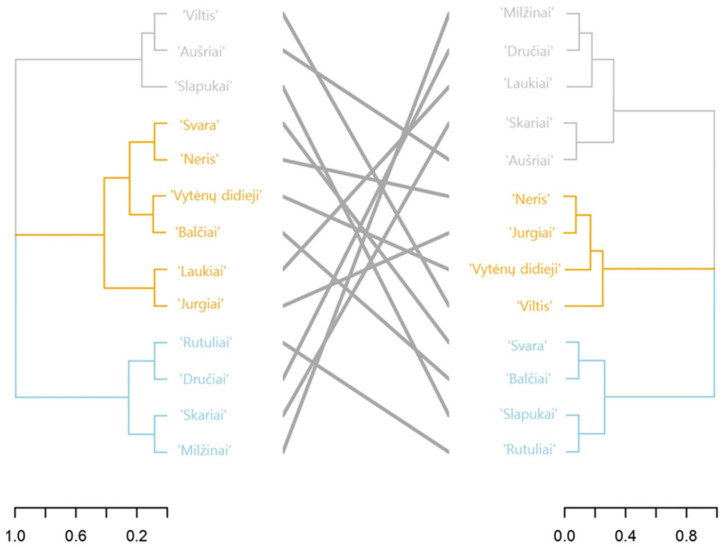
Tanglegram for genetic relations of 13 Lithuanian tomato varieties based on the UPGMA analysis with 12 morphological traits (left) and 7 SSR primers (right). The scale bar indicates the distances between tomato varieties. In the middle, the grey lines indicate the strongness of the relation between morphological and molecular data.

UPGMA cluster analysis was performed using 12 morphological traits and seven SSR primers to assess the genetic diversity of tomato varieties. In the dendrogram and tanglegram, the varieties were divided into three main groups (Figure 2). The cluster of varieties according to morphological traits does not correspond to clustering according to molecular data. According to the grouping of varieties in both dendrograms, the closest relations between morphological and molecular data are for varieties ‘Vytėnų didieji’, ‘Neris’, ‘Aušriai’, ‘Balčiai’ and ‘Rutuliai’. 

**Figure 3:**
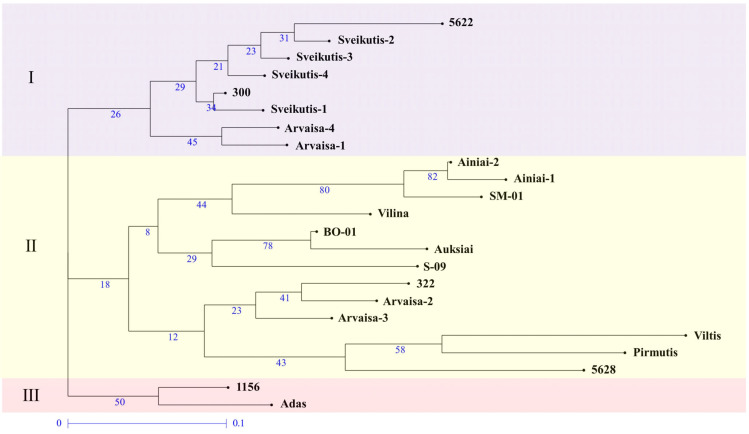
Genetic relations of Lithuanian tomato hybrids and their parental forms based on the UPGMA analysis with 7 SSR primers. Hybrids with the same name but different numbers have more than one molecular fingerprint. The numbers under the branches indicate the percentage of 10,000 bootstrap replications. The scale bar indicates the length of the branches (genetic distance between tomato varieties).

In the UPGMA dendrogram, the Lithuanian tomato hybrids and their parental forms were grouped into three main groups (Figure 3). The grouping of hybrids is related to the parental forms; all genotypes of hybrid ‘Sveikutis’ were grouped in the same group in tandem with both parental forms, namely 5622 and 300 (Table 1). Two hybrid ‘Arvaisa’ genotypes were separated by having the same parental form 300 in the same group. Two other genotypes of hybrids ‘Arvaisa’ belonged to the second group of dendrogram as the molecular fingerprints were more similar to the maternal parental form 322. In this group, one subgroup consisted of hybrid ‘Pirmutis’ and both parental forms 5628 and ‘Viltis’. Two other subgroups of the second cluster were built consistently of (1) hybrid ‘Auksiai’ and parental forms BO-01 and S-09, and (2) both genotypes of hybrid ‘Ainiai’ and parental forms ‘Vilina’ and SM01. The last group of dendrogram consisted of separated hybrid ‘Adas’ with the maternal form 1156.

## 4. Discussion

The fruit size and fruit quantity in the truss are determined by plant genotype [31,32]. Tomato fruit size results from the combination of cell number and cell size, which are determined by both cell division and expansion. Both cell division and cell expansion are under the control of complex interactions between hormone signalling and carbon partitioning, which establish crucial determinants of the quality of ripe fruit, such as the final size, weight, shape, and organoleptic and nutritional traits [33]. Our research has confirmed the findings of previous studies that the genotype of the plant is crucially essential to the size and quantity of fruit on the plant. It is helpful to have the molecular markers, which are necessary to describe the variety, linked to the different traits of the morphological description of the tomato genotypes. Such studies were carried out to identify SSR markers for fruit quality [34], soluble solids [35], drought tolerance [36] and yield-associated traits [37].

An elongated fruit shape was a prevalent morphological feature distinguishing many cultivated varieties from undomesticated accessions. Moreover, SSR markers can be used to distinguish tomato market classes [1,38] between tomatoes’ modern varieties and their landraces [11] or to identify mislabelling in commercially processed tomato products [16]. The major loci identified as contributing to an elongated shape in tomatoes are *sun*, *ovate* and *fs8.1*. Previous phenotypic evaluations demonstrated that three loci regulate unique ovary and fruit elongation aspects in different temporal manners. The most potent effect on organ shape is caused by *sun.* In addition to fruit shape, the *sun* also affected leaf and sepal elongation and stem thickness. 

The synergistic interaction between *sun* and *ovate* or *fs8.1* suggested that the pathways involving *sun*, *ovate* and the gene(s) underlying *fs8.1* may converge at a common node [33,39]. Meanwhile, most of our investigated tomatoes had slightly flattened fruits, and only several had distinguished features with obovoid, round or circular fruit shapes. Only one tomato hybrid ‘Auksiai’ had orange-coloured fruits, while all the rest of the tomato fruits of investigated varieties and hybrids were red-coloured. Geographic origin, cultivation status, fruit size, shape, and growth type were found to exhibit significant genetic differentiation by using SSR markers [17].

Tomato plants’ vegetation period from germination to fruit maturity is a crucial factor in tomato breeding. Usually, the tomato plant vegetation period depends on the variety type and its adaptation in a growing climatic zone. Most Lithuanian tomato varieties are early or medium early and intended for non-heated greenhouses or open fields [40]. In previous studies, scientists observed a positive correlation between the vegetation period and yield: the earlier variety is less productive compared with later ones [40,41]. However, under Lithuanian climatic conditions, late tomato varieties usually fail to mature good quality fruits, so late tomato varieties can be grown only in heated greenhouses.

SSR markers occur frequently in eukaryotic genomes and are highly polymorphic. As a result of their robustness and repeatability, microsatellite markers are widely used codominant markers that have benefits over other molecular markers. SSR analysis offers helpful data for genotyping specific plants or varieties and determining the genetic relatedness of different accessions. For genetic analysis of Lithuanian tomato varieties and hybrids, in total, 14 previously published SSR markers were chosen for the analysis according to the high allelic number and primer informativeness content values. However, only seven of them were suitable for Lithuanian genotypes. Either no fragments were amplificated, or they were monomorphic. One primer, Tom-144, published as very polymorphic and linked to Fusarium wilt resistance for tomato varieties in India [12], was monomorphic for all Lithuanian genotypes. 

Heterozygosity is one of the most used measures for describing genetic diversity and describing studied genotypes [42]. No heterozygosity was observed in the investigated cultivars as they were all homozygous with all the loci used. This shows the high level of purity of these varieties. The hybrids’ average heterozygosity was 0.34, with the highest level in SSR primer TGS0007. The expected heterozygosity for cultivars and hybrids was at the same level (0.51), but the range of values was higher in cultivars, showing the possible influence of inbreeding for hybrids. 

The polymorphism information content (*PIC*) is one of the most important indicators as it gives a reasonable indication of the informativeness of the primers and thus allows for the comparison of results between different laboratories [25]. The average *PIC* values for varieties and hybrids were informative, with values of 0.45 and 0.47, respectively. The most informative SSR primers for varieties were TMS52, TGS0007, LEMDDNa and Tom236-237, and the most informative SSR primers for hybrids were SSR248 and TMS52. The informativeness of other SSR primers is similar to that of other authors [27,28]. Still, there is some tendency for Turkey [38] and Korea [43] to have *PIC* values greater than for Lithuanian genotypes. This indicates that the gene pool of tomato genotypes in Lithuania may be too poor, and there is a need to introduce more germplasm for the creation of new genetic diversity. 

UPGMA cluster analysis demonstrated the usefulness of molecular markers for tomato breeding. They can be used to accurately trace the origin of hybrids and to accurately select the seedlings for tomato breeding. SSR markers will help test seed purity and identify varieties. 

Although the first SSR markers for tomatoes were developed more than three decades ago [30], they are still beneficial for the investigation of the genetic diversity of tomatoes in different geographical regions [35,38,44,45,46] to verify the origin of varieties or link to different traits of fruit [34,35,37] or resistance to biotic and abiotic stresses [12,36,47]. Several hundreds of tomato SSR molecular markers are published and/or placed in the database (http://www.kazusa.or.jp/tomato/) (accessed on 31 July 2024). Paradoxically, no single set of SSR primers is suitable for testing all tomato genotypes at least for one continent. Such sets exist for other horticultural plants like cherry [48] or plum [49] in Europe. In the future, it would be great if such a unique primer set for tomatoes could be established through collaboration between laboratories in different countries.

## 5. Conclusions

This investigation offers valuable insights for characterising cultivated tomato genetic resources, indicating the strong differentiation of phenotypic traits and tomato types. The genetic diversity of 13 Lithuanian tomato varieties, six hybrids and ten parental forms was analysed for seven SSR markers, and it was found that the varieties are homozygous at all loci. The most informative SSR primer pairs for identifying Lithuanian tomato varieties are TMS52, TGS0007, LEMDDNa and Tom236-237. All parental forms of the hybrids were monomorphic at the loci studied, while the hybrids were heterozygous at some loci, inheriting a different allele from each parental form. The most suitable SSR primer pairs for identifying tomato hybrids are SSR248 and TMS52, and for their parental forms, TMS52, SSR248, and TGS0007. Discrepancies in the grouping of tomato varieties according to morphological and molecular data show the need for molecular fingerprinting of tomato varieties to not lose the Lithuanian tomato germplasm by morphological selection.

## Figures and Tables

**Table 1:** Genotypes of tomatoes developed in Lithuania.

No.	Varieties	Parental Forms of Varieties
1.	‘Dručiai’	Nr. 1404/79 × ‘Nina’
2.	‘Vytėnų didieji’	‘Patriot’ × ‘Talaliehin 186’
3.	‘Neris’	‘Dotnuvos tobulybė’ × ‘Grosse fleischige’
4.	‘Milžinai’	Nr. 478/7 × unknown
5.	‘Jurgiai’	Nr.13 × ‘Gribovo gruntiniai’ 1180
6.	‘Aušriai’	‘Jurgiai’ × Nr. 1154
7.	‘Slapukai’	‘Jurgiai’ × ‘Daltona
8.	‘Laukiai’	‘Marcanto’ × ‘Viltis’
9.	‘Svara’	‘Grif’ × ‘Silvana’
10.	‘Balčiai’	‘Grif’ × ‘Silvana’
11.	‘Rutuliai’	‘Šagane’ × ‘Aušriai’
12.	‘Skariai’	‘Everest’ × unknown
13.	‘Viltis’	‘Roma’ × ‘Alpatjevo štambiniai’
	Hybrids	Parental forms of hybrids
1.	‘Sveikutis’	5622 × 300
2.	‘Pirmutis’	5628 × ‘Viltis’
3.	‘Ainiai’	‘Vilina’ × SM-01
4.	‘Adas’	1156 × S-09
5.	‘Auksiai’	BO-01 × S-09
6.	‘Arvaisa’	322 × 300

**Table 3:** Tomato phenotypic traits of different varieties and hybrids.

	Average Fruit Mass, g	Average Fruit Number on Plant	Plant Growth Type ^a^	Leaf Division of Blade ^b^	Peduncle Abscission Layer	Fruit Shape in Longitudinal Section ^c^	Time of Maturity ^d^	Fruit Colour at Maturity	Green Shoulder	Number of Locules	Fruit Firmness ^e^
Varieties											
‘Dručiai’	90 ± 8.6	40 ± 4.6	Ind.	Pin.	Yes	S.F	AE	Red	No	≥6	H
‘Vytėnų didieji’	74 ± 7.4	36 ± 4.5	Det.	Pin.	Yes	S.F	AE	Red	Yes	3–4	H
‘Neris’	75 ± 4.6	42 ± 3.7	Det.	Pin.	No	S.F	AE	Red	No	3–4	M
‘Milžinai’	160 ± 9.6	24 ± 3.2	Ind.	Bip.	Yes	S.F	AE	Red	Yes	≥6	M
‘Jurgiai’	84 ± 6.1	32 ± 3.6	Det.	Bip.	Yes	C	AE	Red	No	2–3	S
‘Aušriai’	92 ± 12.3	30 ± 3.8	Det.	Pin.	Yes	S.F	E	Red	No	4–6	M
‘Slapukai’	90 ± 7.3	33 ± 4.9	Det.	Pin.	Yes	S.F	AE	Red	Yes	3–4	M
‘Laukiai’	60 ± 6.7	39 ± 4.1	Det.	Pin.	Yes	S.F	E	Red	Yes	2–3	S
‘Svara’	60 ± 10.2	65 ± 8.1	Det.	Pin.	Yes	R	L	Red	No	3–4	M
‘Balčiai’	60 ± 6.7	43 ± 4.5	Det.	Bip.	Yes	S.F	AE	Red	No	3–4	H
‘Rutuliai’	101 ± 5.4	37 ± 3.1	Ind.	Pin.	Yes	S.F	AE	Red	No	3–4	M
‘Skariai’	142 ± 14.7	25 ± 5.8	Ind.	Pin.	Yes	O	AE	Red	Yes	2–3	M
‘Viltis’	115 ± 21.2	20 ± 4.1	Det.	Pin.	Yes	S.F	E	Red	Yes	4–5	M
Hybrids											
‘Sveikutis’	78 ± 6.4	23 ± 4.1	Ind.	Pin.	Yes	S.F	AE	Red	No	3–4	M
‘Pirmutis’	74 ± 7.0	24 ± 3.9	Det.	Pin.	Yes	S.F	E	Red	Yes	3–4	M
‘Ainiai’	67 ± 5.9	65 ± 6.1	Det.	Pin.	Yes	O	AE	Red	Yes	2–3	H
‘Adas’	35 ± 4.7	120 ± 9.6	Ind.	Pin.	Yes	R	E	Red	No	3–4	M
‘Auksiai’	38 ± 3.6	111 ± 9.4	Ind.	Pin.	Yes	R	E	Orange	No	2–3	S
‘Arvaisa’	77 ± 5.7	21 ± 4.4	Ind.	Pin.	Yes	S.F	AE	Red	Yes	4–6	M

^a^ Plant growth type: Ind.—indeterminate; Det.—determinate. ^b^ Leaf division of blade: Pin.—pinnate; Bip.—bipinnate. ^c^ Fruit shape in longitudinal section: C—circural; O—obovoid; R—round; S.F.—slightly flattened. ^d^ Time of maturity: E—early; AE—average early; L—late. ^e^ Fruit firmness: H—hard; M—medium; S—soft.

**Table 4:** Molecular profiles of Lithuanian tomato varieties based on SSR markers.

Varieties	SSR-47	Tom 236-237	LEat014	TMS52	SSR248	LEMDDNa	TGS0007
‘Dručiai’	192	176	209	158	247	228	276
‘Vytėnų didieji’	192	190	209	147	245	228	278
‘Neris’	192	190	209	147	247	228	280
‘Milžinai’	192	176	209	158	247	210	276
‘Jurgiai’	192	190	209	147	247	228	278
‘Aušriai’	192	192	209	150	250	228	278
‘Slapukai’	192	190	233	150	245	210	287
‘Skariai’	192	176	209	158	250	212	276
‘Rutuliai’	192	190	233	161	247	212	278
‘Laukiai’	192	176	209	147	247	215	285
‘Svara’	194	190	209	158	247	213	287
‘Balčiai’	192	190	209	158	247	210	285
‘Viltis’	192	180	209	154	247	228	- *

* No fragment amplification.

**Table 5:** Molecular profiles of tomato hybrids and their parental forms based on SSR markers.

Hybrids	SSR-47	Tom236-237	LEat014	TMS52	SSR248	LEMDDNa	TGS0007
‘Sveikutis’	176:192; 176; 192	176:192; 176; 192	233	150	255	213	276:285; 276
‘Pirmutis’	192	180:192;	209:233	154:158	247	213:228; 228	278:285
‘Ainiai’	192	176	209	154:158; 158	247:255	210	276:283
‘Adas’	192:194	176	209	150	247:255	213	276:288
‘Auksiai’	192:194	176	209	154	245:252	213	276
‘Arvaisa’	192	176	209:233; 209; 233	150:154; 150; 154	250:255; 255; 250	213:228; 213; 228	276:285; 276
Parental Forms	SSR-47	Tom236-237	LEat014	TMS52	SSR248	LEMDDNa	TGS0007
5622	192	176	233	150	255	213	285
300	192	176	233	150	255	213	276
5628	176	192	233	158	247	228	285
‘Viltis’	192	180	209	154	247	228	- *
‘Vilina’	192	213	209	158	255	213	276
SM-01	192	176	209	158	245	210	283
1156	192	176	209	150	255	213	288
S-09	194	176	209	161	247	213	276
BO-01	192	176	209	154	252	213	276
322	192	176	233	154	250	228	276

* No fragment amplification.

**Table 6:** SSR primer informativeness for tomato varieties and hybrids.

Varieties					Hybrids					
SSR Primers	Allele Size Range (bp)	No of Allele	*H_e_* *	*H_o_* *	*PIC* *	Allele Size Range (bp)	No of Allele	*H_e_*	*H_o_*	*PIC*
Tom236-237	176–192	4	0.60	0	0.54	176–213	4	0.33	0.31	0.31
SSR-47	192–194	2	0.14	0	0.13	176–194	3	0.38	0.46	0.34
LEMDDNa	210–228	4	0.67	0	0.62	210–228	3	0.54	0.15	0.48
LEat014	209–233	2	0.26	0	0.23	209–233	2	0.50	0.15	0.37
TMS52	147–161	5	0.72	0	0.68	150–161	4	0.63	0.23	0.55
SSR248	245–250	3	0.47	0	0.43	245–255	5	0.66	0.46	0.61
TGS0007	276–287	4	0.71	0	0.66	276–288	5	0.53	0.62	0.49
Mean	-	3	0.51	0	0.47	-	3.71	0.51	0.34	0.45

* *H_e_*—expected heterozygosity; *H_o_*—observed heterozygosity; *PIC*—polymorphism information content.

## Data Availability

Data is contained within the article.
